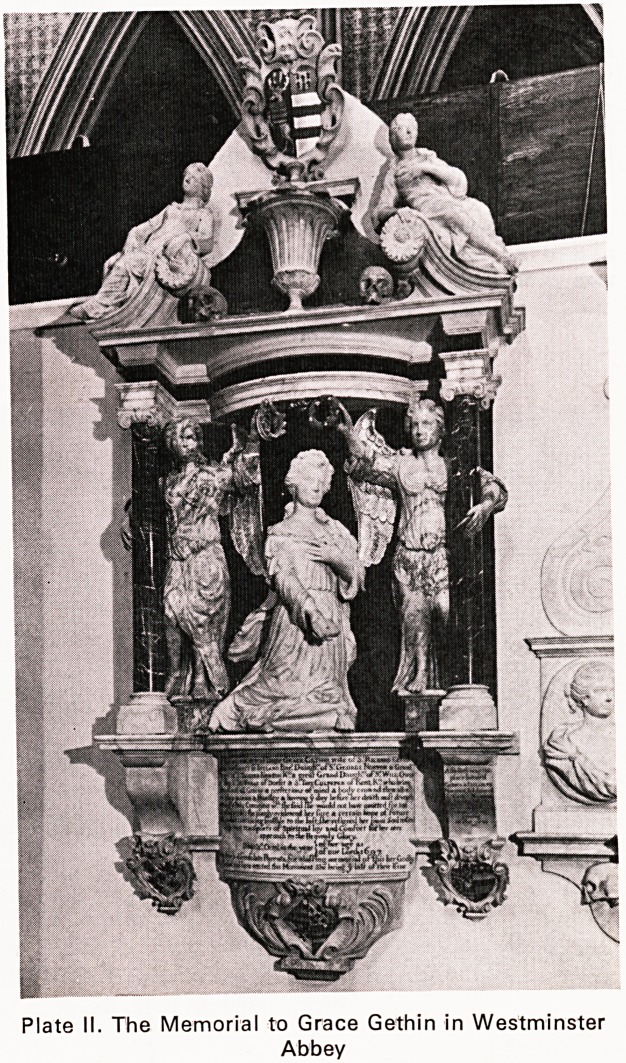# The Gethin Shilling

**Published:** 1974-01

**Authors:** J. Jancar

**Affiliations:** Consultant Psychiatrist, Stoke Park Hospital Group, Bristol


### The Gethin Shilling

J. Jancar, M.B., B.Ch., B.A.O., F.R.C.Psych., D.P.M.
Consultant Psychiatrist, Stoke Park Hospital Group, Bristol

What is the link between Leigh Court Hospital, Westminster Abbey and the North Library of the British Museum? The answer is — Dame Grace Gethin, the last surviving member of the Norton family from Leigh Court, who died in 1697 at the age of 21.

Leigh Court was called Lege in the Domesday Book. It records—"Turstin holds Lege, his father held it in King Edward's time and paid gheld for one hide . . . ." At the Conquest, the Manor was given to the Bishop of Contanus, after whose death William Rufus granted the Manor to Robert FitzHammon, whose daughter married Robert Earl of Gloucester. The Earl of Gloucester sold the Manor of Leigh to Robert FitzHarding, who in 1148 bestowed it to the Abbey of St. Augustine, which he had founded in Bristol. After the dissolution of the Monastery in 1583, it was passed to Paul Bush the first Bishop of Bristol, and afterwards by grant of the King to Sir George Norton. On 16th September, 1651 the Nortons gave shelter for four nights to King Charles II, after his defeat at Worcester. The property passed by marriage to the Trenchard family and it was purchased in the early 19th century by Mr. Philip John Miles who, between 1814 and 1816, built the Leigh Court of today. In January, 1884 the late King Edward VII, then Prince of Wales, was entertained by the late Sir Philip Miles at Leigh Court. In 1917 Leigh Court was bought by the Rev. H. N. Burden to house mentally retarded patients—the function of which is preserved to date. (Jancar, 1969)

The bestowal of the "Gethin Shilling" on a number of widows at Westminster Abbey in a Lenten ceremony was founded by the parents of Dame Grace Gethin, who was married to Sir Richard Gethin, baronet. The terms of the original benefaction are given in a document in Westminster Abbey (W.A.M. 36687) "This is an agreement between the Dean and Chapter of Westminster on the one hand and "Sir George Norton of Abbots Leigh (Plate I) in the County of Somersett Knt. and Dame Frances his wife" on the other, that the "said Dean and Chapter will upon every Ash Wednesday yearely and every yeare for ever cause One Anniversary Sermon to be preached . . . in the said Collegiate Church . . . in the Fore-noone of the same day by some one of the Prebendary's of the said

Plate I. Abbots Leigh Court in 1788 from a drawing by J. H. Grim

Church to be nominated . . . . by the Deane . . . . for the time being in which sermon there shall be a Commemoration of the Vertuous Lady Dame Grace Gething the late wife of S$^{r}$. Richard Gething Barron$^{t}$. and Daughter of the said Sr. George Norton and Dame Frances his wife. And further that they the said Deane and Chapter and their Successors shall . . . . upon the same day in every yeare forever after divine service and the preaching and performing of the Sermon aforementioned give and deliver . . . . unto Forty poor persons inhabiting within the Parish of St. Margaret in Westm$^{r}$. aforesaid to be nominated by the Deane of the said Church for the time being . . . . Twenty Poor men and Twenty Poor women Forty Six-penny Loaves of good weight . . . ."

The Sermon and Commemoration of Dame Grace still continue but the bread charity has ceased. The latest Treasurers' Accounts, dating from the 1850s, record payments for the Bread Money and to the preacher of the Sermon. The money for the poor is given with other charity monies to the Holders of the Dean's Gift. (Mac Michael, 1972)

The circumstance that she was the last surviving member of the Nortons, made her death at the early age of 21 a profound tragedy for her parents and their friends. In the south aisle of Westminster Abbey stands an elaborate memorial to Grace Gethin (Plate II). The inscription reads:

> "To the pious memory of Dame Grace Gethin, wife of S$^{r}$. Richard Gethin of Gethin-Grott in Ireland Bar$^{t}$. Daughter of S$^{r}$. George Norton & Grand Daughter of S$^{r}$. George Norton K$^{ts}$. & Great Grand Daughter of S$^{r}$. Will Owen of Salop S$^{r}$. Tho, Freak of Dorset & S$^{r}$. Tho: Culpeper of Kent, K$^{ts}$. who being adorn'd with all Graces & perfections of mind & body Crowned them all with exemplary patience and Humility & haveing ye day before her death most devoutly receiv'd ye Holy Communion w$^{ch}$. she said she would not have omitted for ten thousand worlds she plainly evidenced her sure & certain hope of future bliss & thus continuing sensible to the last, she resigned her Pious Soul to God in fervent transports of Spiritual Joy and Comfort for her neer approach to the Heavenly Glory.
>
> Obijt. 11th Octo$^{b}$. in the year of our Lord, 1697, of her age 21. her dear & disconsolate parents, for alasting memorial of this her Godly & blessed end have erected this monument she being ye last of their issue.
>
> ---
>
> Also lieth near this place interr'd George and Elizabeth Norton, Children of S$^{r}$. George Norton K$^{t}$. by his wife, Dame Frances Norton both of them dying young in the year."

and leaves no doubt that Grace's outstanding quality was piety. Therefore it seems to be fitting that the small statue incorporated in the memorial should depict the young lady in a kneeling position with a book in her right hand. However, the right arm is extended downwards, carrying the book away from the body, not in a position associated with the act of praying. The answer to the existence of this marble book lies in the British Museum in the North Library, where the rarer books are kept. It is a slim volume published in 1700, after Grace Gethin's death, and its authorship is attributed to her. Under the title Reliquiae Gethinanea, the book is presented as "Some remains of the most ingenious and excellent lady, Grace Lady Gethin, lately deceased; being a collection of choice discources and witty sentences, written for the most part by way of essay at spare hours" and "published by her nearest relations to preserve her memory."

Isaac D'Israeli, scholarly father of Queen Victoria's favourite statesman, had an irresistible urge to collect curiosities presented by literature. Early in the 19th century, alerted by the marble book at Westminster, he traced the real book and was quick to recognise Bacon's writings in it. He did not locate all that was Bacon's, but suspected among what was left unacknowledged quotations from the works of Owen Feltham and Francis Osborne. D'Israeli considered further investigation unnecessary. There can be little doubt that the existence of the book was due simply to

Plate II. The Memorial to Grace Gethin in Westminster Abbey

Grace Gethin's habit of copying passages that had impressed her in books that she read, so that she might the better remember them. After her death, numerous loose sheets were found covered with her handwriting, and on these the presumption of authorship was founded. Therefore Grace Gethin was never party to such gross plagiarism. She was the victim of the excessive devotion and the limited literacy of her parents and friends. (Taylor, 1967).

Because of her relationship with the Culpepers, Dame Grace Gethin has another monument in Hollingbourne Church, near Maidstone in Kent.

Abbots Leigh—Leigh Court Hospital as it is known today—witnessed through its existence many historical events in and around Bristol. It gave refuge to a King, played host to another and to members of the nobility and churchmen. Through Dame Grace Gethin's piety and love of books and poetry, Leigh Court Hospital also secured for itself a permanent monument in Westminster Abbey and in the British Museum.

**Acknowledgements**

I am very grateful to the Keeper of Muniments, of Westminster Abbey, N. H. MacMichael, Esq., for relevant information about Dame Grace Gethin and the charity in memory of her, and for the photograph of the monument to her in the Abbey and to Mrs. K. J. Hiscock for the secretarial work.

**References**

1. JANCAR, J. (1969) Sixty Years of Stoke Park Hospital (1909-1969). This Journal, **84,** 77.
2. MACMICHAEL, N. H. (1972) Keeper of the Muniments, Westminster Abbey. Personal communication.
3. TAYLOR, H. A. (1967) A Young Lady with a Marble Book. Country Life. March 23rd, p. 644.

## Figures and Tables

**Plate I. f1:**
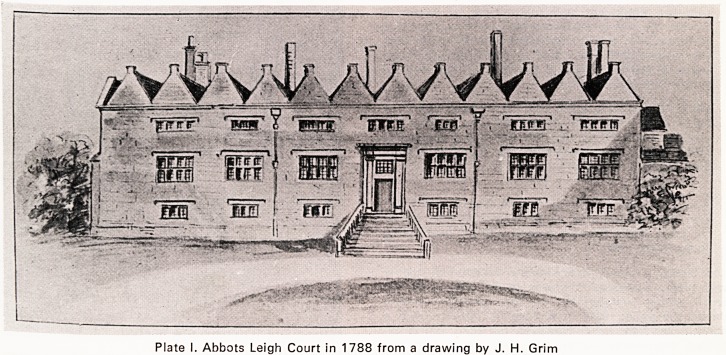


**Plate II. f2:**